# Peroxisome Proliferator-Activated Receptors in HCV-Related Infection

**DOI:** 10.1155/2009/357204

**Published:** 2009-03-30

**Authors:** Sébastien Dharancy, Maud Lemoine, Philippe Mathurin, Lawrence Serfaty, Laurent Dubuquoy

**Affiliations:** ^1^Unité INSERM 795, Université Lille 2, Service des Maladies de l'Appareil Digestif et de la Nutrition, Hôpital Huriez, CHRU Lille, 59037 Lille, France; ^2^Unité INSERM 680, Faculté de Médecine, Université Pierre et Marie Curie, Service d'Hépatologie, Hôpital Saint-Antoine, Assistance Publique - Hôpitaux de Paris (AP-HP), 75012 Paris, France

## Abstract

The topic of peroxisome proliferator-activated receptors has been developed in the field of hepatology allowing envisaging therapeutic strategies for the most frequent chronic liver diseases such as chronic infection with hepatitis C virus (HCV). PPARs contribute to wide physiological processes within the liver such as lipid/glucid metabolisms, inflammatory response, cell differentiation, and cell cycle. In vitro experiments and animal studies showed that PPAR*α* discloses anti-inflammatory property, and PPAR*γ* discloses anti-inflammatory, antifibrogenic, and antiproliferative properties in the liver. Experimental and human studies showed impaired PPARs expression and function during HCV infection. The available nonhepatotoxic agonists of PPARs may constitute a progress in the therapeutic management of patients chronically infected with HCV.

## 1. Introduction

Chronic infection with the hepatitis C virus (HCV) is a major cause of chronic liver disease worldwide, affecting about 3% of the population [1]. The natural history of HCV infection is characterized by a high rate of progression to chronic hepatitis leading, in at least 20% of cases, to cirrhosis and ultimately to hepatocellular carcinoma [2, 3]. The precise mechanisms underlying HCV-related liver injury are not well understood but involve a cell-mediated immune response with lymphocytic infiltration leading to a chronic inflammatory response and progressive scale fibrosis which are dependent on proinflammatory and fibrogenic mediators. HCV infection is also characterized by disruption of lipid and glucid metabolisms leading to hepatocyte fat accumulation (also called hepatic steatosis) and an increase risk of diabetes [4–7]. These latter are under dependence, at least in part, of peroxisome proliferator-activated receptors functions.

Peroxisome proliferator-activated receptors (PPARs) belong to the nuclear receptor superfamily and require heterodimerization with receptor X for retinoids (RXR) in order to function [8]. The first PPAR identified was murine PPAR*α* [9]. Since its description in 1990, a family of homologous receptors has been identified in various species. The PPAR family includes three subtypes: PPAR*α* (NR1C1), PPAR*β* (PPAR*δ* or NR1C2), and PPAR*γ* (NR1C3) [10]. PPAR*α*/*γ*, together with their obligate partner RXR, are the three main nuclear receptors expressed in the liver. These receptors contribute to the great diversity of physiological processes in the liver, such as control of lipid and glucid metabolisms, inflammatory responses, and cellular differentiation and proliferation. PPAR activation has been associated with anti-inflammatory and antifibrotic functions in the liver. In light of their multiple activities, PPARs quickly became considered as therapeutic targets of the most widespread human metabolic diseases (obesity, diabetes, and atherosclerosis). The development of new nonhepatotoxic ligands made it possible to use PPARs as new therapeutic targets in hepatology. For these reasons, recent discoveries in the field of PPARs are of intense interest not only to fundamental researchers but to clinicians as well. This review will first describe the main functions of PPARs within the liver. Then the interactions between PPARs and HCV will be developed to provide a precise picture of the potential role of PPARs in HCV pathophysiology and therapy.

## 2. Control of Lipid and Lipoprotein Metabolism

PPAR*α* is strongly involved in the control of lipid and lipoprotein metabolisms. PPAR*α* ^−/−^ mice display minimal accumulation of triglycerides in the liver under fed conditions, but manifest an exaggerated steatotic response to fasting [11]. Moreover, PPAR*α* ^−/−^ mice fed a high-fat diet showed massive accumulation of lipids in the liver, highlighting its crucial role in lipid metabolism [12]. Intracellular fatty acid (FA) concentrations are controlled, in part, by regulation of the FA import and export system. PPARs control the uptake of FA in liver; for example, PPAR agonists control expression of the FA transport protein (FATP-1) responsible for FA transport across the cell membrane, FA translocase (FAT/CD36), and the hepatic cytosolic FA binding protein (L-FABP) involved in FA trafficking [13]. The absence of induction of FATP-1 and FAT/CD36 mRNA in liver by PPAR*α* activators has been well demonstrated in PPAR*α* ^−/−^ mice. PPAR*α* and -*γ* also prevent the efflux of FA by promoting their activation into fatty acyl CoA thioesters by the acyl-CoA synthetase ACS [14, 15]. In addition, PPAR*α* stimulates activated FA catabolism by the peroxisomal, microsomal, and mitochondrial *β*-oxidation systems. It regulates the expression of genes coding for enzymes such as acyl-CoA oxidase (AOX), the first and main enzyme in the classic peroxisomal *β*-oxidation system. It also activates the transcription of genes encoding for classical microsomal *β*-oxidation system cytochrome P450 CYP4A (A1 and A3) isoforms, and stimulates activated FA catabolism by mitochondrial *β*-oxidation. It is generally accepted that oxidation of long-chain FA is regulated at the level of the liver carnitine palmitoyl-transferase I (LCPT1), whose product catalyzes the initial step in long-chain FA import into mitochondria. PPAR*α* has been demonstrated to affect FA import into mitochondria by upregulating expression of the LCPT1 gene [14]. Concerning PPAR*γ*, treatment of HepG2 with troglitazone, a synthetic agonist, specifically increased recycling by peroxisomal *β*-oxidation of C18 to C16 FAs, and the interconversion of long-chain FAs was associated with reduced de novo lipogenesis. It is conceivable that these effects of troglitazone on oxidation, interconversion, and synthesis-saturated FA play a major role in cellular energy metabolism, membrane lipid composition and turnover [16].

It has been convincingly established that PPAR*α* activation increases plasma HDL cholesterol and enhances reverse cholesterol transport via the induction of hepatic apolipoprotein A-I and apolipoprotein A-II expression in human liver. Nevertheless, PPAR*α* activators affect HDL metabolism in an opposite manner in rodents and humans. While fibrate treatment of rats lowers plasma HDL, an increase is generally observed in humans; such an increase in HDL plasma levels is related, at least in part, to changes in apoAI and apoA-II gene expression in liver. ApoA-I and apoA-II gene transcription is upregulated in humans and repressed in rodents by PPAR*α* activators. Treatment of human primary hepatocytes with fibrates increases mRNA levels and secretion of apoA-I. Moreover, in patients with familial combined hyperlipoproteinemia, ciprofibrate administration enhances production of apoA-I [17].

## 3. Anti-Inflammatory and Immunomodulatory Properties of PPAR in the Liver

PPAR*α* exert catabolic function through peroxisomal, microsomal, and mitochondrial *β*-oxydation pathways, and therefore allows the use of FA by hepatocytes as energetic substrate. Thus, it degrades several lipid inflammatory mediators (prostaglandins, leukotriens) using these metabolic pathways [18, 19]. There are numerous experimental and clinical evidences in favor of anti-inflammatory activity of PPAR in the liver. PPAR*α* and PPAR*γ* were shown to negatively interfere with the nuclear factor (NF)-*κ*B, signal transducers and activators of transcription (STAT), and activating protein 1 (AP-1) signalling pathways in primary hepatocyte cultures and monocytes/macrophages [20, 21]. Delerive et al. showed that fibrates, which are the main synthetic agonists of PPAR*α* induced the expression of I*κ*B*α* protein in primary human hepatocytes, whereas neither I*κ*B kinase activity nor the degradation rate of I*κ*B*α* was affected. The consequence is that the downstream inflammatory responses genes such as IL-2, IL-6, IL-8, TNF*α*, and metalloproteases are inhibited [20]. PPAR*α* can also control hepatic inflammation by regulating hemostatic factors and acute-phase response proteins in hepatocytes [22].

T-lymphocytes, natural killer cells, and monocytes/macrophages express significant amount of PPAR*α* and *γ*when activated, and they inhibit the production of inflammatory and immunomodulatory activity cytokines [23–28]. In lymphocytes, immunomodulatory activity is supported by a decrease in nuclear factor of activated T-cells (NFAT) activity which regulate IL-2 promoter [23, 29]. PPAR*γ* inhibit the proliferation of T-lymphocytes and the production of IFN*γ*, TNF*α*, and IL-2 [30].

Animal studies confirmed the role of PPAR*α* and PPAR*γ* in inflammation control showing that PPAR*α* ^−/−^ mice display a prolonged response to inflammation induced by arachidonic acid or zymosan [31–33]. Interestingly, PPAR*α* ^−/−^ and PPAR*γ* ^+/−^ mice displayed an exacerbated sensibility to hepatitis in different models of liver injury [34, 35].

## 4. Antifibrogenic Activities of PPAR in the Liver

Hepatic stellate cells (HSCs) are the main cells responsible for liver modulation, by producing the protein of the extracellular matrix [36, 37]. In response to liver injury, HSC changes from a quiescent to an activated phenotype. This activation process includes a phenotypic change to a myofibroblast-like cell, an increased proliferation rate, loss of retinoid stores, and increased production of extracellular matrix proteins, chemokines, cytokines, and contractility. Interestingly, HSC activation is associated with impaired expression of PPAR*γ* and with a decrease in binding to the peroxisome proliferator response element (PPRE) in vivo, whereas NF*κ*B and AP-1 binding is increased. HSC activation is reversed by treatment with PPAR*γ* agonists in vitro, suggesting antifibrogenic activity [38, 39]. PPAR*γ* activation dose-dependently inhibited HSC proliferation and chemotaxis induced by platelet-derived growth factor. This effect was independent of changes in postreceptor signalling or expression of c-fos and c-myc, and was associated with inhibition of cell progression beyond the G1 phase. PPAR*γ* activation also resulted in complete inhibition of the expression of monocyte chemotactic protein (MCP1), a potent chemoattractant for monocytes and T-lymphocytes [40]. All things considered, PPAR*γ* activation in vitro resulted in decreases in HSC proliferation, migration, and chemokine expression, three pivotal actions relevant to the process of liver wound healing and fibrogenesis. A study performed in a model of liver fibrosis induced by dimethylnitrosamine or carbon tetrachloride showed that oral administration of pioglitazone or rosiglitazone reduces hepatic extracellular matrix deposition and HSC activation [41]. PPAR*α* ligands may also have rescue effects on hepatic fibrosis. Using thioacetamide models of liver cirrhosis, animals treated with a diet containing one of the two PPAR*α* ligands, Wy-14 643 (WY) or fenofibrate, displayed reduced hepatic fibrosis. PPAR*α* ligands had an antifibrotic action in the thioacetamine model of liver cirrhosis, probably due to an antioxidant effect of enhanced catalase expression and activity in the liver [42].

## 5. Interaction between HCV and PPARs

Liver inflammation, hepatocyte fat accumulation, and diabetes are three pivotal hallmarks in the natural history of chronic HCV infection which are at least in part controlled by PPARs. Thus, it was tempting for several teams to investigate whether PPAR*α*/*γ* may be impaired and/or nonfunctional, and might therefore play a role in the physiopathology of HCV infection.

Numerous studies have demonstrated that PPAR*α*/*γ* were physiologically highly expressed by parenchymal hepatic cells, where they play a pivotal anti-inflammatory and metabolic role. Several teams have demonstrated, using in vitroand ex vivo approaches, a decrease expression and functional activity of PPAR*α* in hepatocytes during HCV infection, which may contribute to the pathogenesis of the disease. (1) During chronic HCV infection in humans [43, 44], (2) in an experimental model of transgenic mouse [4], and (3) in transfected hepatocytes expressing the capsid protein of HCV [4, 43, 44]. These abnormalities may be the consequence of viral capsid inhibiting transcriptional activity of genes involved in lipid metabolism of infected host cell [4, 43, 44].

In the same line, De Gottardi et al. showed that (1) PPAR*γ* expression was significantly lower in genotype 3 compared with genotype 1 HCV infection and (2) steatosis was associated to decreased levels of PPAR*γ* in genotype 1 HCV infection. In this study, there was no significant relationship between PPARs mRNA levels and liver activity or fibrosis. The presences of steatosis and hepatitis C virus genotype 3 were both associated with a significant downregulation of PPARs [44].

The ways in which impaired expression of PPAR*α*/*γ* contribute to the pathogenesis of HCV hepatitis are numerous. As previously described, PPAR*α* activators play a regulatory role in the inflammatory response at the hepatic level by inhibiting cytokines and acute-phase inflammatory protein production by negatively interfering with NF-*κ*B and AP-1 pathways. Clinical evidence for the anti-inflammatory role of PPAR*α* in the liver was provided by the characterization of PPAR*α* ^−/−^ mice which were more susceptible to various hepatotoxic compounds and had delayed liver regeneration compared to their wild-type littermates. A recent study also demonstrated that activation of PPAR*α* limits the expression of the TH1 cytokine profile and TNF*α* production by human CD4-positive T-cells, a cytokine pattern known to substantially contribute to HCV pathogenesis.

Preliminary human data reinforce interest in this topic. Two pilot studies suggested therapeutic effects of bezafibrate, an agonist of PPAR*α*/*β*/*γ*, during chronic HCV infection. In the first open trial, 30 patients unresponsive to interferon monotherapy were treated for 6 months with 400 mg/day [45]. At endpoint, viral load and liver enzymes were significantly decreased, suggesting anti-inflammatory and antiviral activities of bezafibrate. More recently, 7 patients received 400 mg/day of bezafibrate for 8 weeks. Once again, ALT, AST, and viral load were lowered compared to baseline [46]. Taken together, these results may argue in favor of restoration of transcriptional activity of the hepatoprotective PPAR*α* and/or PPAR*γ* receptors during chronic HCV infection. Pioglitazone, an agonist of PPAR*γ*, is currently evaluated in patient with chronic HCV infection and insulin resistance (http://clinicaltrials.gov/show/NCT00189163).

## 6. Conclusion

Chronic infection with HCV is a major cause of chronic liver disease worldwide and is associated with chronic liver inflammation, hepatocyte fat accumulation, and diabetes. Nuclear hormone receptors PPAR*α* and *γ* are involved in the transduction of metabolic and nutritional signals into transcriptional responses in order to maintain liver homeostasis. Evidence exists in favor of a decreased expression and functional activity of PPAR*α*/*γ* in hepatocytes during HCV infection related to HCV core protein, suggesting that HCV may have evolved strategies to prevent inducing, or to overcome, an efficient response by the host. This decreased expression of PPAR*α*/*γ* may be involved in the pathogenesis of HCV infection through an alteration of the protective effects of these nuclear receptors against hepatic inflammation and fibrosis. Taken together, all these considerations suggest that PPAR*α*/*γ* may represent new potential therapeutic targets in HCV infection.

## Nomenclature:

PPAR: Peroxisome proliferator-activated receptor
RXR: Retinoid X receptor
HCV: Hepatitis C virus
FA: Fatty acid.
